# Sensing and Processing for Infrared Vision: Methods and Applications

**DOI:** 10.3390/s23073764

**Published:** 2023-04-06

**Authors:** Saed Moradi

**Affiliations:** 1 Department of Electrical and Computer Engineering, Faculty of Science and Engineering, Laval University, Quebec City, QC G1V 0A6, Canada; saed.moradi.1@ulaval.ca; 2 Computer Vision and Systems Laboratory (CVSL), Laval University, Quebec City, QC G1V 0A6, Canada

Dear readers and fellow researchers,

I am pleased to introduce this Special Issue of *Sensors*, entitled “Sensing and Processing for Infrared Vision: Methods and Applications”. As the Guest Editor of this Special Issue, it has been an honor to work with a group of distinguished experts in the field to present to you this collection of high-quality research articles.

This Special Issue covers a broad range of topics in infrared (IR) vision technology, including novel sensing techniques, advanced signal processing algorithms, and innovative applications. The papers featured in this Special Issue provide valuable insights into the state-of-the-art research in the field and demonstrate the potential of IR vision technology in a range of applications, such as surveillance and remote sensing.

The papers in this Special Issue are as follows:

1. **Fractal Texture Enhancement of Simulated Infrared Images Using a CNN-Based Neural Style Transfer Algorithm with a Histogram Matching Technique [1]:** This paper proposes a novel convolutional neural network (CNN)-based infrared image enhancement method to transform pseudo-realistic regions of simulation-based infrared images into real infrared texture.
2. **Identity-Preserved Human Posture Detection in Infrared Thermal Images: A Benchmark [2]:** This paper presents a novel task called Identity-Preserved Human Posture Detection in Thermal images (IPHPDT), which fills the gap between human detection and human pose estimation while preserving privacy. This task serves a threefold purpose: firstly, to establish an identity-preserved task using thermal images; secondly, to obtain more information than just the location of persons provided by human detection, thus enhancing computer vision applications; and thirdly, to overcome the challenges of collecting well-annotated data for human pose estimation in thermal images.
3. **Thermal Infrared Tracking Method Based on Efficient Global Information Perception [3]:** This paper proposes a Thermal InfraRed (TIR) tracking method that addresses the issue of limited resistance to occlusion and interference from similar targets in current methods. The proposed method utilizes an efficient global information perception approach, which incorporates a Transformer structure for feature extraction and fusion to effectively obtain global semantic information from images.
4. **Thermal Water Prospection with UAV, Low-Cost Sensors and GIS. Application to the Case of La Hermida [4]:** This paper describes a research project that proposes a methodology for exploring hydrothermal resources in an efficient, cost-effective, and expeditious manner. The methodology involves using photogrammetry techniques along with visual and thermal images captured by UAVs to generate temperature maps or thermal orthomosaics. By analysing these maps using GIS tools, zones with potential geothermal interest along rivers or lakes can be automatically identified.
5. **YOLO-SASE: An Improved YOLO Algorithm for the Small Targets Detection in Complex Backgrounds [5]:** This research paper presents an improved detection algorithm called YOLO-SASE, which aims to enhance the detection ability of infrared small targets in complex backgrounds. The YOLO detection framework and SRGAN network serve as the basis for the algorithm, with super-resolution reconstructed images as the input. The algorithm utilizes the SASE module, SPP module, and multi-level receptive field structure while adjusting the number of detection output layers. This is achieved through exploring feature weight to enhance the feature utilization efficiency. Compared with the original model, the proposed algorithm in this study demonstrated improved accuracy and recall rate.
6. **Low-SNR Infrared Point Target Detection and Tracking via Saliency-Guided Double-Stage Particle Filter [6]:** The objective of this research paper is to propose a saliency-guided double-stage particle filter (SGDS-PF) approach for detecting and tracking targets. The SGDS-PF model is composed of two stages: searching particle filter (PF) and tracking PF. To enhance the targets and suppress noise, single-frame and multi-frame target accumulation methods are introduced before the searching PF. To extract the likelihood saliency and obtain an appropriate proposal density, likelihood estimation filter and image block segmentation are used. The searching PF is guided by this proposal density to detect potential targets efficiently. The tracking PF is then used to track and confirm these potential targets using the results from the searching PF. Finally, the real targets’ path is produced. Compared with existing methods, SGDS-PF optimizes the proposal density for low-SNR images, and can detect potential targets quickly and accurately using a small number of precise particles. In addition, the tracking PF can continue tracking the targets using very few particles, even in the presence of intense noise, initialized by the searching PF. Moreover, the parameters are appropriately chosen through experiments.
7. **Local Spatial–Temporal Matching Method for Space-Based Infrared Aerial Target Detection [7]:** In this research paper, a new concise method for detecting aerial targets is proposed, which is based on local spatial–temporal matching (LSM). LSM involves four main steps: local normalization, local direction matching, spatial–temporal joint modelling, and inverse matching. The local normalization step is intended to amplify the target to the same level as the surrounding clutter, thereby preventing weak targets from being disregarded. Next, a direction-matching technique is used to estimate the motion direction of the background between the basic and reference frames. A spatial–temporal joint model is then constructed to enhance the target and reduce strong clutter. Similarly, inverse matching is carried out to further amplify the target. The output is a salience map, on which the aerial target can be identified through adaptive threshold segmentation.

Together, these papers demonstrate the wide range of research in the field of infrared vision technology, from novel sensing and processing techniques to innovative applications. This Special Issue contributes to advancing the state of the art in IR vision technology, and I hope that it will inspire further research in this important field.
